# Heads, Tails, and Tools: Morphogenesis of a Giant Single-Celled Organism

**DOI:** 10.1371/journal.pbio.1001862

**Published:** 2014-05-13

**Authors:** Mary Hoff

**Affiliations:** Freelance Science Writer, Stillwater, Minnesota, United States of America

**Figure:**
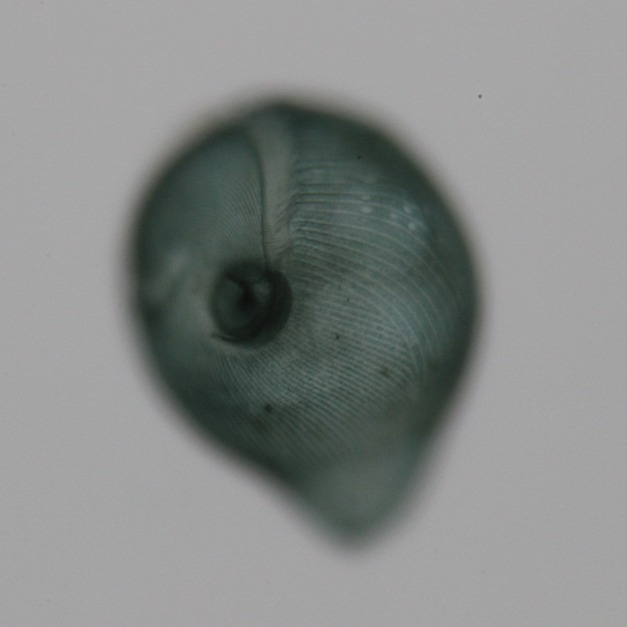
***Stentor coeruleus*** **.** Brightfield image of *Stentor coeruleus* clearly shows the cell's stripes. The feeding organelle, or oral apparatus, is present at the top of the image while the cell anchor is oriented toward the bottom of the image. *Image credit: Mark M. Slabodnick - University of California, San Francisco.*

It's (relatively) easy to conceptualize how multicellular organisms, like plants and people, generate the specialized structures they need for everyday life: cells differentiate to take on different tasks, such as photosynthesizing and breathing. But what about one-celled organisms? How do their components segregate within a single cell to develop head-to-tail polarity and unique structures, such as cilia?

In search of an answer, Mark M. Slabodnick, Wallace F. Marshall, and colleagues turned to *Stentor coeruleus*, a single-celled aquatic filter-feeder that has remarkably distinct body parts, including cilia, an oral apparatus it uses to ingest bacteria, and a foot-like holdfast with which it clings to surfaces. This giant ciliate—it measures close to a millimeter from head to toe—is renowned for its ability to regenerate from just a tiny part of itself. In fact, because its size allowed elegant surgical manipulations, *Stentor* became a classical model organism for studying shape and pattern formation in single-celled organisms in the first half of the 20th century. But somehow *Stentor* fell off the cell biologist's map in the 1970s: it was never developed as a molecular or genetic model system, and work on the organism largely stopped at that time. Slabodnick, Marshall, and colleagues now put *Stentor* back on the map by providing the first molecular analysis of regeneration in this organism.

These researchers began by testing whether RNA interference (RNAi) could be used to study *Stentor* morphogenesis. RNAi is a common experimental tool that exploits natural regulatory pathways, allowing the silencing of specific gene transcripts in order to discern how the proteins they encode work. Normally these pathways use small endogenous RNA molecules called microRNAs to target the silencing activity to specific transcripts, but by introducing artificial RNA molecules (siRNAs), scientists can hijack the microRNA system for their own purposes. A look at portions of *Stentor's* genome revealed to the authors that it contains genes that code for proteins needed for the microRNA pathway (such as Argonaute and Dicer proteins). To see if this machinery could function to silence *Stentor's* gene transcripts, as it does in other organisms, the researchers fed to *Stentor* bacteria that produce double-stranded RNA molecules that can give rise to siRNAs targeting a gene that encodes a protein, α-tubulin, involved with shaping cellular structures. If the RNAi process worked as expected, they should see as a result the loss of the protein. Indeed, after several days, the bacteria-fed *Stentor* lost their linear form and became globular. Further analysis using antibodies against α-tubulin confirmed that RNAi had successfully caused the loss of this protein, resulting in the abnormal development of *Stentor* by disrupting the tubulin-mediated organization of the macronucleus and of cell structures called the cortical rows.

Having confirmed that RNAi works in *Stentor*, the research team then used this approach to study this organism's regeneration and shape development. Knowing from others' work that the regeneration of *Stentor's* oral apparatus after injury is similar to oral apparatus generation during cell division, the team homed in on the protein Mob1, which is known to play a role in morphogenesis in multicellular organisms, as a likely candidate for regulating shape development in *Stentor*. Using an antibody to Mob1, they learned that the enzyme concentrates in *Stentor's* tail as well as in the region around the oral apparatus.

To determine whether and how Mob1 is involved in shaping *Stentor*, the team then made bacteria containing RNAi vectors targeting Mob1 and fed them to *Stentor*. Cells that received the RNAi-spiked bacteria lost their wineglass shape, then either became elongated and curved or developed a blob-like shape with multiple posteriors and oral apparatus regeneration sites.

Curious to learn more about the formation of these shapes, the researchers photographed RNAi recipients every two hours after treatment. They found that oral apparatus regeneration was followed by development of a new posterior in the wrong place, and concluded that Mob1 is needed for oral apparatus and posterior structure localization. They also observed that when Mob1 was depleted by RNAi, it was first lost at the mouth end in elongated cells, and later entirely absent as seen in the blob-like cells—suggesting that Mob1 does different jobs in different places.

To examine Mob1's role in regeneration, the researchers fed to *Stentor* the Mob1 RNAi bacteria, then cut them in half and watched the two pieces develop. Anterior segments grew ectopic tails next to previous posterior, while posterior segments grew a new posterior where the oral apparatus normally would develop—suggesting that Mob1 is not needed to start regeneration, but plays an important role in polarized cell growth. They also observed that a small fraction of the cells so treated were able to successfully grow the missing structures (although not in normal proportion), the researchers concluded that proportionality and regeneration are functionally different, with proportionality more sensitive to RNAi knockdown.

Finally, the researchers decided to examine what happens if they cut out parts of the cell where Mob1 localizes shortly after applying RNAi treatment. The treatment essentially sped up knockdown-induced changes, supporting the idea that Mob1 plays a role in both anterior and posterior polarity.

This molecular genetic study of regeneration in *Stentor* brings this formidable single-celled organism back to research life. The authors encourage other researchers interested in development of shape in single cells to take advantage of this model system, and plan to do so themselves as they work to further understand cell polarity, regeneration, and morphogenesis in single-celled organisms.

**Slabodnick MM, Ruby JG, Dunn JG, Feldman JL, DeRisi JL, et al. (2014) The Kinase Regulator Mob1 Acts as a Patterning Protein for** ***Stentor*** **Morphogenesis.** doi:10.1371/journal.pbio.1001861

